# Diabetes Mellitus and Younger Age Are Risk Factors for Hyperphosphatemia in Peritoneal Dialysis Patients

**DOI:** 10.3390/nu9020152

**Published:** 2017-02-17

**Authors:** Rameez Imtiaz, Steven Hawken, Brendan B. McCormick, Simon Leung, Swapnil Hiremath, Deborah L. Zimmerman

**Affiliations:** 1 Department of Medicine, University of Ottawa, Ottawa, ON K1N 6N5, Canada; rimti037@uottawa.ca; 2 School of Epidemiology, Public Health and Preventive Medicine, University of Ottawa, Ottawa Hospital Research Institute, Clinical Epidemiology Program, Ottawa, ON K1Y 4E9, Canada; shawken@ohri.ca; 3 Department of Medicine, Division of Nephrology, Ottawa Hospital, University of Ottawa, Kidney Research Centre, Ottawa Hospital Research Institute, Ottawa, ON K1H 7W9, Canada; shiremath@toh.on.ca (S.H.); dzimmerman@toh.on.ca (D.L.Z.); 4 Ottawa Hospital Academic Family Health Team, Ottawa Hospital, Ottawa, ON K1Y 4K7, Canada; sleung@toh.on.ca

**Keywords:** peritoneal dialysis, hyperphosphatemia, phosphate binders, diabetes mellitus

## Abstract

Hyperphosphatemia has been associated with adverse outcomes in patients with end stage kidney disease (ESKD). The purpose of this study was to determine risk factors for hyperphosphatemia in ESKD patients treated with peritoneal dialysis (PD). This information will be used to develop a patient specific phosphate binder application to facilitate patient self-management of serum phosphate. Adult PD patients documented their food, beverage, and phosphate binder intake for three days using a dietitian developed food journal. Phosphate content of meals was calculated using the ESHA Food Processor SQL Software (ESHA Research, Salem, UT, USA). Clinic biochemistry tests and an adequacy assessment (Baxter Adequest program) were done. Univariate logistic regression was used to determine predictors of serum phosphate >1.78 mmol/L. A multivariable logistic regression model was then fit including those variables that achieved a significance level of *p* < 0.20 in univariate analyses. Sixty patients (38 men, 22 women) completed the protocol; they were 60 ± 17 years old, 50% had a history of diabetes mellitus (DM) and 33% had hyperphosphatemia (PO_4_ > 1.78 mmol/L). In univariate analysis, the variables associated with an increased risk of hyperphosphatemia with a *p*-value < 0.2 were male gender (*p* = 0.13), younger age (0.07), presence of DM (0.005), higher dose of calcium carbonate (0.08), higher parathyroid serum concentration (0.08), lower phosphate intake (0.03), lower measured glomerular filtration rate (0.15), higher phosphate excretion (0.11), and a higher body mass index (0.15). After multivariable logistic regression analysis, younger age (odds ratio (OR) 0.023 per decade, 95% confidence interval (CI) 0.00065 to 0.455; *p* = 0.012), presence of diabetes (OR 11.40, 95 CI 2.82 to 61.55; *p* = 0.0003), and measured GFR (OR 0.052 per mL/min decrease; 95% CI 0.0025 to 0.66) were associated with hyperphosphatemia. Our results support that younger age and diabetes mellitus are significant risk factors for hyperphosphatemia. These findings warrant further investigation to determine the potential mechanisms that predispose younger patients and those with DM to hyperphosphatemia.

## 1. Background

Hyperphosphatemia is associated with adverse outcomes in patients with end stage kidney disease (ESKD) including abnormal bone and mineral metabolism, vascular and soft tissue calcification, and cardiovascular morbidity and mortality [1,2,3,4,5,6]. In an attempt to mitigate this risk, it has been recommended that, when patients have high serum phosphate, that it be lowered towards the normal range but many patients fail to achieve this goal [5,7,8,9,10].

The interventions to control serum phosphate include adequate dialysis, dietary phosphate restriction, and phosphate-binding agents. Unfortunately, the most common dialysis prescriptions provide inadequate phosphorous removal alone such that almost all nutritionally replete patients will be in a positive phosphate balance [11]. Nutritional interventions aim to limit the amount of dietary phosphate to less than 1100 mg per day [7]. However, not all phosphate is equally bioavailable as phosphate salts are much more readily absorbed than the phosphate contained in phytic acid [12]. The phosphate food pyramid is a pictorial representation of six food levels based on their phosphorus content, phosphorus to protein ratio, and phosphorus bioavailability [12]. Very little attention has focused on other factors that might be associated with increased phosphate absorption. Lastly, phosphate binders can be taken with meals to limit intestinal absorption of phosphate. Despite the variability in daily meal phosphate consumption, it is common to prescribe a fixed dose of phosphate binders. This phosphate intake variability may lead to a mismatch between phosphate intake and phosphate binder dose resulting in an increase in phosphate and/or calcium absorption [13].

Based on the premise that self-adjustment of phosphate binders by dialysis patients using a mobile application (APP) may improve phosphate control, the aim of this three-phase study is to develop a novel phosphate counting program for Apple iOS devices (Apple, Cupertino, CA, USA). Phase 1 of the study was to determine the daily meal phosphate variability to ensure that development of such an APP was warranted [13]. The data gathered in Phase 1 was also to be used to develop a patient specific regression equation in which the APP would automatically adjust the amount of phosphate binder to be taken with a meal based on identified risk factors for hyperphosphatemia. Therefore, the purpose of this study was to determine risk factors for hyperphosphatemia using readily available clinical and laboratory variables in this treated peritoneal dialysis patient population.

## 2. Methods

The methods for Phase 1 have been described previously [13]. The study was approved by the Ottawa Health Science Research Ethics Network (approval code 20120105-01H). English or French speaking/writing adult patients who had ESKD treated with peritoneal dialysis and were taking phosphate binder therapy were eligible for participation. Participants were excluded from the study if they (i) were unable or unwilling to give informed consent; (ii) had hypercalcemia; (iii) were visually or hearing impaired; or (iv) were expected to receive a renal transplant during the time of the study. After obtaining informed consent, patients were taught to use a registered dietitian (RD) developed food journal. One week prior to their routine clinic visit, participants were asked to document their food and beverage intake, and the number of phosphate binders taken with meals for three days using the food journals. The phosphate content of the foods and beverages was calculated with ESHA Food Processor SQL Software (ESHA Research, Salem, UT, USA). Other sources were used for foods not found in the software including published manuscripts (USDA National Nutrient Database for Standard Reference, Release 25), government nutrient databases (Food Standards Australia New Zealand’s NUTTAB 2010 Online Searchable Database), and the food manufacturer. Pre-clinical biochemistry tests including serum calcium, phosphate, parathyroid hormone, and a standard adequacy assessment using the Baxter Adequest program (Baxter Healthcare, Deerfield, IL, USA) were performed. Twenty-four hour urinary and dialysate phosphate was also measured in the samples collected for the Adequest. These values were multiplied by three to be consistent with the diet diaries in the tables. The percent absorption of phosphate was estimated from the ((urinary ± dialysate phosphate)/dietary phosphate intake) based on the assumption that patients were in a steady state (not anabolic or catabolic). The diet diaries were reviewed and adjustments made for the bioavailability of phosphate based on the food pyramid as this might have an impact on the percent absorption of phosphate [12]. A phosphate index (PIs) was determined by multiplying the amount of phosphate in a food by the phosphate pyramid rating (based on a foods position in the pyramid; 1–6) as a proportion of the total phosphate intake for the three days. The PIs for all the meals were then added together to generate a summary PI for each patient.

Data including basic patient characteristics and medications was entered into an Excel spreadsheet. Summary descriptive statistics to describe the population were calculated. Univariate logistic regression was then undertaken to determine predictors of hyperphosphatemia (>1.78 mmol/L). Independent variables considered included: age, sex, diabetes, phosphate intake, phosphate binder intake, % phosphate absorption, glomerular filtration rate, dialysate creatinine clearance, dialysate Kt/V, calcitriol dose, serum albumin, and bicarbonate. Serum parathyroid hormone (PTH) concentration was explored as a continuous variable and by quintiles as extremes of PTH may have different effects on serum phosphate. Glomerular filtration rate was estimated from the average of the urinary urea and creatinine clearance. A multivariable logistic regression model was then fit using backwards selection including those variables that achieved a nominal significance level of *p* < 0.20 in the univariate analyses. Variables were retained in the multivariable model regardless of final statistical significance. A post hoc exploratory analysis was undertaken in an attempt to determine the potential mechanism by which diabetes mellitus was found to be a risk factor for hyperphosphatemia. All analyses were with SAS software, version 9.4 (SAS Institute, Cary NC, USA).

## 3. Results

### Participants and Recruitment

A total of 150 patients were screened for the study. Twenty-two patients were not eligible (prior to consent three patients died, nine patients received renal transplants, six patients switched to hemodialysis, two patients recovered renal function, and two patients were not taking a phosphate binder). Fifty patients declined to participate. Seventy-eight peritoneal dialysis patients consented to the study, 60 (38 men and 22 women) completed the study protocol (Table 1). Reasons for participant withdrawal from the study included: three received a kidney transplant, one died, five withdrew consent, seven were unable or unwilling to follow the protocol, and two were found to be ineligible.

On average patients were 60 ± 17 years old, predominately Caucasian and 50% had a history of diabetes mellitus (Table 1). The majority of patients were taking calcium carbonate as their phosphate binder with a median intake of 2000 mg (Interquartile range (IQR) 1166–3500) per day. Only six of the patients were using sevelamer hydrochloride as a phosphate binder. Median daily phosphate intake including snacks was 1008 mg (IQR 818–1252) per day with the majority of phosphate being consumed with supper.

The variables that were associated with an increased risk of phosphate of >1.78 mmol/L were sex, diabetes mellitus, binder dose taken, percent phosphate absorption, and PTH (Table 2). Those factors associated with a decreased risk of phosphate of >1.78 mmol/L included older age, higher phosphate intake and higher GFR (Table 2). In multivariate logistic regression analysis age, diabetes mellitus, and GFR remained statistically significant (Table 3).

Patients with diabetes mellitus eat less phosphate, take more phosphate binders, but excrete more phosphate consistent with higher gastrointestinal absorption of phosphate (Table 4). The estimated percent absorption of phosphate was greater than 50% in 23 of the 60 PD patients. The PI was higher (2.59 vs. 2.28; *p* = 0.03) in the ‘high’ absorbers. Fifty percent of patients with diabetes mellitus (*N* = 15) were high absorbers compared to 27% (*N* = 8) of patients without diabetes mellitus (*p* = 0.06). However, PI (2.33 vs. 2.41, *p* = 0.50) did not differ between patients with diabetes who were and were not high absorbers, suggesting that the mechanism for enhanced gastrointestinal absorption of phosphate differed by diabetes status.

## 4. Discussion

We have shown that diabetes mellitus, younger age, and lower GFR are independent risk factors for hyperphosphatemia in spite of a treatment regimen that includes dietician assessment and counselling every six to eight weeks, adjustment of dialysis prescription, and phosphate binders. These interventions have resulted in the paradoxical association of lower phosphate intake and higher phosphate binder dose in patients with hyperphosphatemia.

Hyperphosphatemia is the result of a complex interplay of multiple variables that includes phosphate intake, phosphate absorption, and phosphate removal. Accurate accounting of phosphate intake is complicated by diet dairy accuracy and hidden phosphate additives. The bioavailability of phosphate can differ dramatically depending on the food source. We have shown a calculated PI that takes phosphate bioavailability into consideration was higher in patients with greater percent phosphate absorption, further supporting this claim. The tremendous inter-patient phosphate absorption variability has been highlighted previously [14]. It is also important to note that many medications contain phosphate and can serve as a significant source of hidden but very bioavailable phosphate [15].

Hyperphosphatemia has been described in animal models of diabetes mellitus and in humans with diabetic nephropathy but the mechanism(s) is unclear [16,17,18]. It is possible that patients with diabetes mellitus simply eat more phosphate or more bioavailable phosphate. The diet diaries do not support an increase in phosphate intake by diabetes status. Nor do patients with diabetes mellitus appear to eat more bioavailable phosphate as demonstrated by the PI values. There may be something unique to the ‘diabetic milieu’ that leads to enhanced phosphate absorption (*p* = 0.06). Very little research has focused on factors that activate/reduce the activity of the sodium phosphate co-transporter in the gut (Na-P-2b). The active form of vitamin D (1,25-dihydroxyvitamin D) has been shown to upregulate the activity of Na-P-2b and thereby enhance absorption of phosphate [19,20]. Almost half of our patients (29/60) were taking calcitriol but the average daily dose was low and may explain the lack of association with hyperphosphatemia. PTH has also been shown to increase intestinal phosphate transport in perfused duodenal loops and has been associated with hyperphosphatemia which appears to be consistent with our results [18]. Nicotinamide has been shown to reduce the activity of Na-P-2b and has been used clinically to reduce serum phosphate concentration for patients treated with hemodialysis [21]. Interestingly, 24,25-dihydroxyvitamin D has also been shown to reduce phosphate transport by blocking the effects of 1,25-dihydroxyvitamin D on Na-P-2b transport activity [20]. Furthermore, patients with diabetes mellitus have been shown to have reduced levels of 24,25-dihydroxyvitamin D [20,22]. This implies that patients with diabetes mellitus treated with calcitriol might be expected to have the highest serum phosphate levels. Lastly, several medications used for the treatment of diabetes mellitus include highly bioavailable inorganic phosphate as an additive which is not captured in the diet diaries but would appear as increased gastrointestinal absorption of phosphate and increase phosphate excretion [15].

The relationship between younger age and hyperphosphatemia has been previously reported in the literature [23]. In animal models, the activity of the Na-P-2b is reduced with increasing age [19]. In humans, lower serum phosphate in older individuals may be the result of multiple factors. With more advanced age, poorer nutritional status is more prevalent [24]. Enzymatic activity that leads to phosphate absorption in the gut may also be reduced in the elderly [25]. The association between preserved renal function and better phosphate control in peritoneal dialysis patients is intuitive and has been shown previously [25,26].

Our study has a number of strengths including the prospective ascertainment of phosphate intake, binder dose consumed, and collection of a large number of selected variables including patient demographics, laboratory tests, and medications. However, there are a number of weaknesses including the relatively small number of patients included in the study and the need to assume that our patients were in steady state with respect to phosphate. In Canada, there is limited access to non-calcium containing phosphate binders and this may limit the generalizability of our results. The accuracy of diet diaries and food phosphate content is always of concern. We cannot exclude the possibility that some patients may not have included all foods/beverages consumed or accurately recorded the number of phosphate binders that were taken. Moreover, the patient population was treated with adjustments to diet, dialysis prescription, and phosphate binders which may have limited our ability to determine all of the predictors of serum phosphate and the strength of the association. Lastly, the method that we used to calculate the phosphate bioavailability has not been validated.

## 5. Conclusions

Our results support the importance of younger age and diabetes mellitus as risk factors for hyperphosphatemia. This finding will have important clinical implications if supported by data from additional studies and will be used in the development of our phosphate binder APP.

## Figures and Tables

**Table 1:** Patient demographics.

Variable	Overall	PO_4_ ≥ 1.78 mmol/L	PO_4_ < 1.78 mmol/L
*N*	60	20	40
Sex (Female)	22	10 (50%)	12 (30%)
Age years (SD)	62.3 ± 13.9	57.8 ± 14.1	64.6 ± 13.4
Race (*N*)	Caucasian (51), Aboriginal (1), African American (3), Asian (5)	Caucasian (17), African American (3)	Caucasian (34), Aboriginal (1), Asian (5)
Etiology of ESKD	DM (18), PCKD (8), GN (6), Other (28)	DM (9), PCKD (4), GN (1), Other (6)	DM (9), PCKD (4), GN (5), Other (22)
Body Mass Index	28.4 ± 5.4	29.8 ± 7.1	27.6 ± 4.3
DM	30 (50%)	15 (75%)	15 (37.5%)
P intake median (IQR)–3 day (mg)	3024.6 (2453.3, 3754.7)	2648.9 (2082.6, 3511.0)	3149.7 (2498.4, 3878.0)
Phosphate Index	2.373 ± 0.419	2.376 ± 0.395	2.372 ± 0.44
P excretion (median, IQR) 3-day (mg)	1387.0 (1096.5, 1858.5)	1710.0 (1159.0, 2128.6)	1373.0 (1072.6, 1697.9)
Total daily Ca intake (median, IQR)	2000 (1166.1, 3500.0)	3000 (1500.0, 4183.3)	1500 (1000, 3000)
% P Absorption	0.54 ± 0.29	0.66 ± 0.35	0.47 ± 0.24
GFR mL/min	4.15 ± 3.2	3.3 ± 3.1	4.6 ± 3.2
Calcitriol Dose (weekly dose, µg)	(*n* = 29) median 0.75, range 0.75–3	(*n* = 10) median 1.25, range 0.75–3	(*n* = 19) median 0.75, range 0.75–3
Serum Ca (mmol/L)	2.3 ± 0.15	2.3 ± 0.16	2.3 ± 0.15
Serum Alb (g/L)	31.4 ± 3.9	31.2±4.4	31.6 ± 3.6
Serum HCO_3_ (mmol/L)	24.6 ± 6	23.8± 3.0	25.1 ± 3.1
Serum PO_4_ (mmol/L)	1.7 ± 0.45	2.2 ± 0.3	1.44 ± 0.25
PTH (pmol/L)	34.57 ± 34.65	43.2 ± 34.5	30.2 ± 34.3
PTH by Quintile			
Q1	12	2	10
Q2	12	4	8
Q3	12	4	8
Q4	12	4	8
Q5	12	6	6
Dialysate CrCl (weekly)	34.77 ± 15.56	36.89 ± 17.87	33.61 ± 14.39
Dialysate Kt/V (weekly)	1.33 ± 0.56	1.406 ± 0.62	1.29 ± 0.53

*N* = number, SD = standard deviation, ESKD = End Stage Kidney Disease, DM = diabetes mellitus, PCKD = polycystic kidney disease, GN = glomerulonephritis, PO_4_ = phosphate, IQR = interquartile range, mg = milligrams, GFR = glomerular filtration rate, mL/min = millilitres per minute, µg = microgram, Ca = calcium, mmol/L = millimole per litre, Alb = albumin, g = gram, HCO_3_ = bicarbonate, PTH = parathyroid hormone, pmol = picomole, CrCl = creatinine clearance, Kt/V = urea clearance, time, volume.

**Table 2:** Risk factors for a serum phosphate >1.78 mmol/L, univariate.

Variable	OR	95% CI Lower	Upper	*p* Value
Age years (per decade)	0.69	0.45	1.03	0.07
Gender (Female)	0.43	0.14	1.29	0.13
Diabetes Mellitus	5.00	1.59	18.03	0.005
Body Mass Index	5.82	0.53	75.90	0.15
P intake (mg)	0.05	0.0002	0.72	0.03
PO_4_ excretion (mg)	5.63	0.68	52.86	0.11
Ca carbonate intake (mg)	9.20	0.78	129.37	0.08
Serum Ca (mmol/L)	4.61	0.32	78.68	0.26
Serum PTH (pmol/L)	10.18	0.35	657.13	0.18
Serum PTH (by quintile)	0.75	0.50	1.11	0.15
Serum Alb (g/L)	1.03	0.89	1.19	0.69
Serum HCO_3_ (mmol/L)	0.87	0.72	1.04	0.15
GFR (mL/min)	0.22	0.024	1.66	0.15
Calcitriol (µg)	1.70	0.21	12.96	0.60
Dialysate Kt/V	2.24	0.26	21.05	0.46
Dialysate CrCl	2.84	0.20	43.71	0.44

PO_4_ = phosphate, mg = milligrams, Ca = calcium, mmol/L = millimole per litre, PTH = parathyroid hormone, pmol = picomole, Alb = albumin, g = gram, HCO_3_ = bicarbonate, GFR = glomerular filtration rate, mL/min = millilitres per minute, µg = micrograms, Kt/V = urea clearance, time, volume, CrCL = creatinine clearance.

**Table 3:** Risk factors for a serum phosphate > 1.78 mmol/L, multivariable model.

Variable	Adjusted OR	95% CI Lower	Upper	*p* Value
Age (per decade)	0.023	0.00065	0.455	0.012
DM	11.40	2.82	61.55	0.0003
GFR (per mL/min)	0.052	0.0025	0.66	0.022

DM = diabetes mellitus, GFR = glomerular filtration rate, mL/min = millilitres per minute.

**Table 4:** Post-hoc analysis of differences between patients with and without diabetes mellitus.

Variable	No DM	DM	*p* Value
*N*	30	30	
Women	12 (40%)	10 (40%)	0.79
Age years (SD)	60.9 ± 15.7	63.7 ± 12.0	0.44
Body Mass Index	26.7 ± 3.8	29. 9 ± 6.3	0.02
PO_4_ > 1.78 mmol/L	5	15	0.006
P intake median (IQR), 3-day (mg)	3048.1 (2473.1, 3726.6)	2980.3 (2307.8, 4098.1)	0.71
Phosphate Index	2.410 ± 0.474	2.336 ± 0.367	0.503
PO_4_ excretion (median, IQR), 3-day (mg)	1296 (1059.4, 1660.6)	1627.9 (1163.3, 2102.4)	0.035
Total daily Ca carbonate intake (median, IQR) (mg)	1500 (958.3, 2750)	2875 (1333.3, 4045.8)	0.08
% Phosphate Absorption	0.48 ± 0.24	0.59 ± 0.33	0.13
GFR mL/min	3.7 ± 3.4	4.7 ± 3.0	0.23
Calcitriol Dose (weekly dose, µg)	*N* = 15 Median 0.75 (0.75, 1.5)	*N* = 13 Median 0.75 (0.75, 1.5)	0.6
Serum Ca (mmol/L)	2.3 ± 0.15	2.3 ± 0.16	0.98
Serum PO_4_ (mmol/L)	1.6 ± 0.3	1.8 ± 0.5	0.06
PTH (pmol/L)	34.4 ± 38.6	34.8 ± 30.9	0.96
<15 pmol/L (*N* = 15)	7	8	
>100 pmol/L (*N* = 3)	1	2	
Dialysate CrCL (weekly)	33.7 ± 15.1	35.7 ± 16.2	0.64
Dialysate Kt/V (weekly)	1.36 ± 0.6	1.3 ± 0.6	0.73

DM = diabetes mellitus, *N* = number, SD = standard deviation, PO_4_ = phosphate, mmol/L = millimole per litre, IQR = interquartile range, mg = milligrams, GFR = glomerular filtration rate, mL/min = millilitres per minute, µg = microgram, Ca = calcium, Alb = albumin, g = gram, HCO_3_ = bicarbonate, PTH = parathyroid hormone, pmol = picomole, CrCl = creatinine clearance, Kt/V = urea clearance, time, volume.
